# Natural Contamination with Mycotoxins Produced by *Fusarium graminearum* and *Fusarium poae* in Malting Barley in Argentina

**DOI:** 10.3390/toxins10020078

**Published:** 2018-02-11

**Authors:** María Soledad Nogueira, Julieta Decundo, Mauro Martinez, Susana Nelly Dieguez, Federico Moreyra, Maria Virginia Moreno, Sebastian Alberto Stenglein

**Affiliations:** 1 Laboratorio de Biología Funcional y Biotecnología (BIOLAB), UNCPBA-CICBA, INBIOTEC-CONICET, Av. República de Italia 780, Azul, 7300 Buenos Aires, Argentina; solenogueira@gmail.com (M.S.N.); maurom@faa.unicen.edu.ar (M.M.); vmoreno@faa.unicen.edu.ar (M.V.M.); 2 Área de Toxicología, Departamento de Fisiopatología, Centro de Investigación Veterinaria de Tandil (CIVETAN) CONICET-CICBA, Facultad de Ciencias Veterinarias-UNCPBA, Campus Universitario, Paraje Arroyo Seco s/n, Tandil, 7000 Buenos Aires, Argentina; jdecundo@vet.unicen.edu.ar (J.D.); susadie@vet.unicen.edu.ar (S.N.D.); 3 Estación Experimental Agropecuaria INTA Bordenave, Ruta Provincial 76 Km 36,5, Bordenave, 8187 Buenos Aires, Argentina; moreyra.federico@inta.gob.ar; 4 Área de Microbiología, Facultad de Agronomía de Azul-UNCPBA, Av. República de Italia 780, Azul, 7300 Buenos Aires, Argentina

**Keywords:** Fusarium graminearum, Fusarium poae, DON, NIV, barley

## Abstract

Two of the most common species of toxin-producing *Fusarium* contaminating small cereal grains are *Fusarium graminearum* and *F. poae*; with both elaborating diverse toxins, especially deoxynivalenol (DON) and nivalenol (NIV), respectively. The objective of our work during the 2012–2014 growing seasons was to screen crops for the most commonly isolated *Fusarium* species and to quantify DON and NIV toxins in natural malting-barley samples from different producing areas of Argentina. We identified 1180 *Fusarium* isolates in the 119 samples analyzed, with 51.2% being *F. graminearum*, 26.2% *F. poae* and 22.6% other species. We found high concentrations of mycotoxins, at maximum values of 12 μg/g of DON and 7.71 μg/g of NIV. Of the samples, 23% exhibited DON at an average of 2.36 μg/g, with 44% exceeding the maximum limits (average of 5.24 μg/g); 29% contained NIV at an average of 2.36 μg/g; 7% contained both DON and NIV; and 55% were without DON or NIV. Finally, we report the mycotoxin contamination of the grain samples produced by *F. graminearum* and *F. poae*, those being the most frequent *Fusarium* species present. We identified the main *Fusarium* species affecting natural malting-barley grains in Argentina and documented the presence of many samples with elevated concentrations of DON and NIV. To our knowledge, the investigation reported here was the first to quantify the contamination by *Fusarium* and its toxins in natural samples of malting barley in Argentina.

## 1. Introduction

Barley (*Hordeum vulgare* L.)—one of the most commercially significant cereals worldwide—is grown in certain regions of Africa and in the highlands of Asia and Latin America, where the grain is usually consumed for human nutrition. In Europe, barley is used for animal feed and in the preparation of alcoholic beverages such as beer and whisky. In Argentina, the grain’s main destination is likewise the brewing industry, although the use of barley for animal feed is increasing. The total world production of barley in 2016 was about 150 million tons [1], with the US Department of Agriculture (USDA) estimating that the figure for 2016/2017 will be about 144 million tons. The main barley producers are the European Union (at 59.8 million tons) and Russia (at 17.5 million tons), with Argentina being the tenth worldwide (at 3.2 million tons) (https://www.produccionmundialcebada.com/). According to the Ministry of Agroindustry of Argentina [2], the barley-cultivated area devoted to the brewing industry in 2015/2016 was 1.47 million ha, mainly localized in the central/southern area of the Buenos Aires province (±90%), an increase of 33.6% over the previous growing season.

Barley, along with other cereals, can be contaminated by pathogenic and/or nonpathogenic fungi, and a high level of contamination with fungal species such as *Fusarium* and/or *Penicillium* on barley grains has evidenced a significant correlation with a low quality of the resulting malts [3]. A complex of toxigenic and nontoxigenic *Fusarium* species has been isolated from cereals (wheat, barley, and oat) with symptoms of Fusarium head blight (FHB), a major disease of small-grain cereals that reduces seed quality and yield [4,5]. Moreover, certain *Fusarium* species are potential producers of the trichothecenes deoxynivalenol (DON) and nivalenol (NIV), toxins that are hazardous to the health of both humans and animals through association with certain pathologies—e.g., emesis and feed refusal or loss of appetite, among other negative consequences [4,6].

In Europe, cereal grains are frequently contaminated by *Fusarium* species such as *F. culmorum*, *F. avenaceum*, and *F. poae*; however, generally, the most common contaminant is *F. graminearum* [7]. The occurrence of *Fusarium* species, however, is subject to climatic changes over a period of years and to agricultural practices, such as pesticide applications and nitrogen fertilization [8,9,10]. In Sweden, *F. avenaceum* was the species with highest incidence on wheat kernels, followed by *F. poae*, whereas *F. graminearum* was the most abundant [9]. In the Umbria region, in central Italy, Beccari et al. [11] found that the predominant *Fusarium* species on malting-barley grains was *F. avenaceum*, followed by *F. graminearum* and *F. poae*. In a three-year field study, *F. avenaceum* (in 2012) and *F. poae* (in 2013)—but never *F. graminearum*—were the most frequently isolated species from barley grains [8].

As with the occurrence of *Fusarium* species, the mycotoxin content on small-grain cereals has been found to vary. DON levels were seen to have increased after a warm and intense rainfall had occurred during or after anthesis [12,13]. Moreover, recently, Li et al. [14,15] demonstrated the presence of a gene that converts DON to DON-3-*O*-glucoside (nontoxic) and NIV to NIV-*O*-β-d-glucoside (nontoxic), thus effecting a detoxification.

*Fusarium graminearum* and *F. poae* are typical type-B-trichothecene producers, the former being the greater DON producer and the latter the greater NIV producer [10,16,17,18]. Certain studies, however, have also cited *F. poae* as a type-A-trichothecene (HT-2 and T-2) producer [19].

Although neither the number of *Fusarium*-species isolates nor the quantity of fungal DNA in any specific one necessarily indicates a high concentration of a specific toxin in the resulting cereal samples, and vice versa, various studies have described significant correlations between these variables. *F. graminearum* and *F. culmorum* DNAs have been positively associated with the occurrence of DON, *F. poae* DNAs with NIV, and *F. langsethiae* DNAs with HT-2 and T-2 in barley samples [20]; *F. graminearum* with DON in wheat [21,22]; and *F. graminearum* with DON as well as *F. langsethiae* with both HT-2 and T-2 in oat [23].

Specific regulations for processed and unprocessed cereal grains with respect to mycotoxin concentrations could vary between regions and/or countries. In the European Union, the European Commission has set maximum limits for unprocessed grains (other than durum wheat, oats, and maize) at 1.25 μg/g (ppm) of DON; however, the maximum limit for NIV has not yet been established, the latter being more toxic than the former [24]. By contrast, in Brazil, the Health Surveillance Agency for Brazil (Agência Nacional de Vigilância Sanitària: ANVISA), established a limit of 1.00 μg/g of DON for malted barley [25]. The limits for DON and/or NIV contamination, however, have not yet been established in certain countries of South America, such as Argentina.

Studies on natural samples in different countries—for example, the UK—have indicated that barley grains are contaminated mainly with DON, followed by NIV, although in most instances the levels have been below the established legislative thresholds [20]. In Brazil, 9 out of 50 barley-grain samples were detected with DON in 2013 (ranging from 0.2 to 15.1 μg/g), while 292 barley samples from a seven-year survey were found to contain a high level of DON in Uruguay (at an average of 2.76 μg/g) [26,27].

Many studies have demonstrated a decrease in the quality of different cereals as a result of the presence of *Fusarium* species, even in the absence of contamination from the mycotoxins produced by this fungal genus; with wheat being the most extensively studied cereal at the global and national level [16,22,28]. Little is known, however, about the prevalence and diversity of *Fusarium* species or the accompanying presence of their mycotoxins through field studies on barley [8,20], mainly because the investigations at characterizing the *Fusarium graminearum*-species complex and its potential to produce toxins have been conducted in vitro [29,30]. Although few such studies have been performed on barley, the presence of *Fusarium* species on this cereal is known to affect different parameters for malting—e.g., the germination capacity of the grain and the increase in protein and nitrogen content [20,31,32]. Many agrarian nations have found an increasing need to understand more completely the impact of the presence of *Fusarium* on grain yield and quality and the effects of the associated mycotoxins on humans and animals. Moreover, a more thorough knowledge of the presence of *Fusarium* species and their associated toxins in cereal grains will influence the nature of commercial regulations and modify the thresholds for toxin tolerance on a worldwide basis.

The aim of this work was therefore to study the occurrence of *Fusarium* species—with particular attention to *F. graminearum* and *F. poae*—in malting-barley samples from the principal producing areas of Argentina and to quantify the levels of the toxins DON and NIV contaminating the grains.

## 2. Results

### 2.1. Fusarium-Species Identification

*Fusarium graminearum* and *F. poae* were the most frequently isolated *Fusarium* species among all the samples analyzed. We identified morphologically a total of 1180 *Fusarium* isolates, where 51.2% were designated as *F. graminearum*, 26.2% as *F. poae*, and 22.6% as other species—i.e., 6.5% as *F. incarnatum*–*F. equiseti* species complex, 5.6% as *F. chlamydosporum*, 3.6% as *F. oxysporum*, 2.5% as *F. acuminatum*, 2.2% as *F. proliferatum*, 1.4% as *F. tricinctum*, 0.6% as *F. cerealis*, and 0.2% as *F. pseudograminearum*.

**Figure 1:**
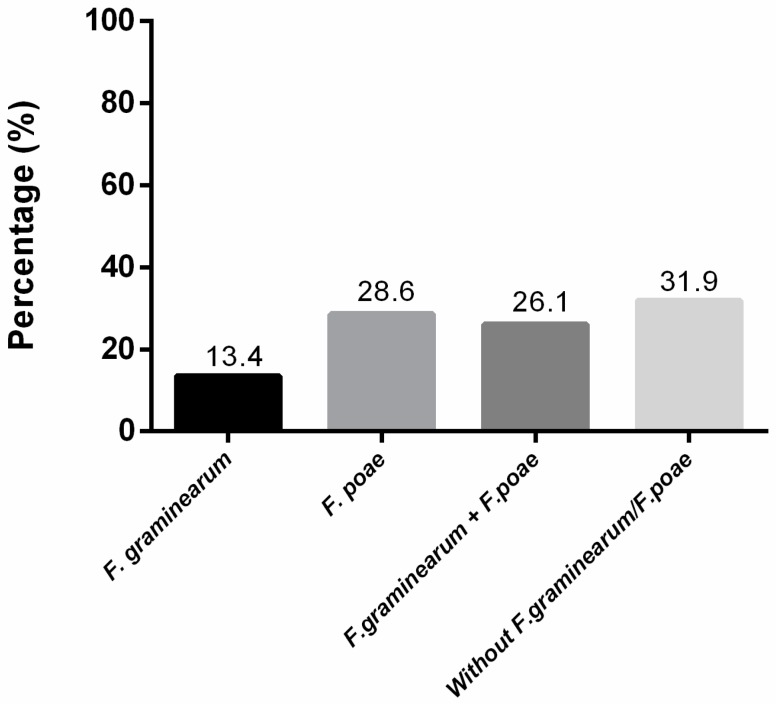
Percentage of samples with or without *Fusarium graminearum* and/or *F. poae*. In the figure, the percentage detected among all the isolates is plotted on the *ordinate* for each of the *Fusarium* species or their combination indicated on the abscissa. The precise percentages of the different species or combinations are listed above the respective bars.

*Fusarium graminearum* isolates were obtained in 13.4% of the total of barley samples analyzed, with each year varying as follows (Table S1; Figure 1): 22.7% (2012), 37.7% (2013), and 52.8% (2014). Conversely, *F. poae* was isolated in 28.6% of the total samples, with annual variations at: 68.2% (2012), 37.7% (2013) and 75.0% (2014). In 26.1% of the total samples, both *F. graminearum* and *F. poae* were present, whereas in only 38 out of the 119 samples (31.9%) was neither *F. graminearum* nor *F. poae* isolated (Figure 1).

Among the 24 localities sampled, in only one was neither *F. graminearum* nor *F. poae* isolated. In contrast, *F. graminearum* was isolated from 20 different localities and *F. poae* from 23 (Table S1).

All the barley genotypes sampled were hosts to *F. graminearum* and/or *F. poae* (Table S1).

From all the *F. graminearum* and *F. poae* DNA isolates used for molecular characterization, the respective corresponding fragments of ≈400 bp and ≈220 bp could be amplified. Moreover, from the *F. graminearum* DNAs, a fragment of 282 bp was amplified that corresponded to the DON genotype and, from the *F. poae* DNAs, a fragment of 296 bp corresponding to that of NIV.

Although *F. graminearum* and *F. poae* were typically respective DON and NIV producers, we could isolate two more *Fusarium* species capable of producing these toxins, *F. pseudograminearum* (DON) and *F. cerealis* (NIV); however, these species were obtained sporadically—*F. cerealis* from only Samples 14, 22, 35, 89, 90, and 96 (one contaminated grain per sample) and *F. pseudograminearum* from only Samples 16 and 23 (one contaminated grain per sample).

### 2.2. Trichothecene Screening

**Figure 2:**
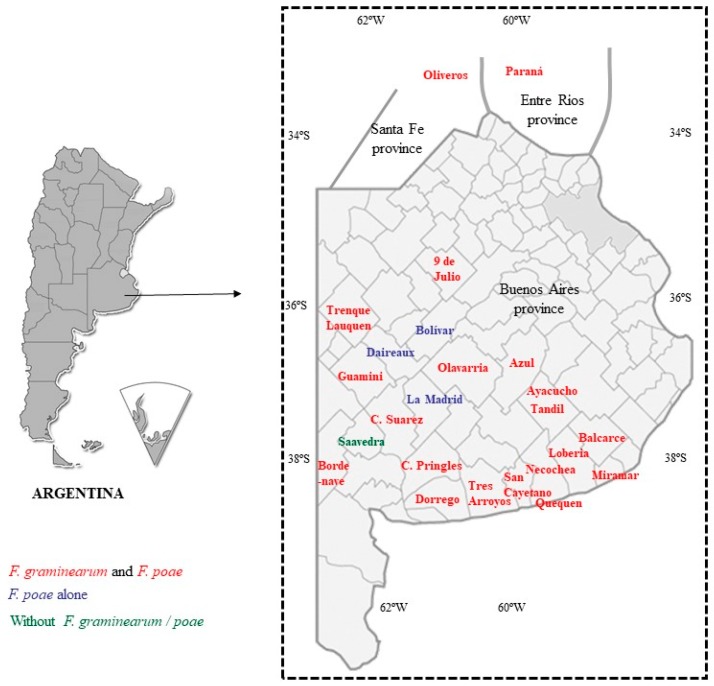
Localities sampled and the presence of *Fusarium graminearum* and/or *F. poae*. In the figure, the left panel shows Argentina in its entirety, including the territory in Antartica (pie-shaped inset); the right panel depicts the Buenos Aires province (arrow in left panel). Color key to *Fusarium* detection in the map of the right panel: red, both *F. graminearum* and *F. poae*; blue, *F. poae* alone; green, without *F. graminearum*/*F. poae*.

**Figure 3:**
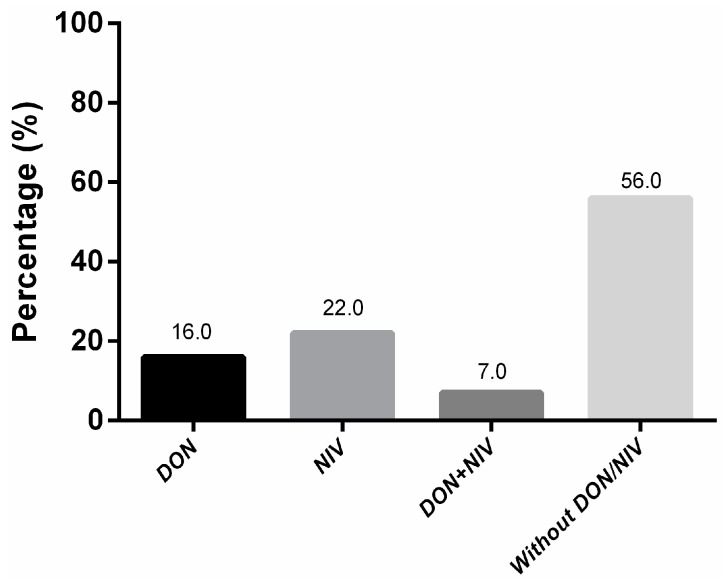
Percentage of samples with or without deoxynivalenol (DON) and/or nivalenol (NIV). In the figure, the percentage of trichothecene detected among all the samples analyzed is plotted on the ordinate of DON alone (black bar), NIV alone (medium-gray bar), both DON and NIV (dark-gray bar), and neither DON nor NIV (light-gray bar) as indicated on the abscissa. The precise percentages of the samples with either or both toxins, or with neither are listed above the respective bars.

From all the samples analyzed, 16% (19/119) contained DON (average of 2.6 μg/g), 44% of which group exceeded the maximum limits (average of 5.24 μg/g). A barley sample (Sample 62, Table S1) from Miramar (on the coast of the Buenos Aires province) exhibited the highest level of DON (12 μg/g; Table S1; Figure 2). Twenty-two percent of the samples were contaminated with NIV (average of 2.36 μg/g), and one sample (Sample 117, Table S1) from Paraná (Entre Ríos province; Figure 2) contained the highest concentration (7.71 μg/g). Seven percent of the samples exhibited DON and NIV in combination, whereas 55% of the samples were free of both toxins (Table S1; Figure 3).

A large fraction of the samples (45%) contained at least one of the two trichothecenes, with 2014 having a significantly higher percentage of samples with toxins than either 2012 or 2013 (Table 1). Moreover, except for 2014, where the percentage of each toxin was equal, NIV occurred in a higher percent of the samples than did DON (Table 1).

Among the localities sampled, in only three were neither DON nor NIV detected (Oliveros, 9 de Julio, and Daireaux; Table S1; Figure 2), whereas DON was a contaminant in 12 different localities and NIV in 19 (Table S1). DON and/or NIV were found in all the sampled barley genotypes.

### 2.3. Statistical Analyses

The frequencies of *F. graminearum* and *F. poae* were 0.85 and 0.92, respectively. The correlation coefficients between the percent presence of *F. graminearum* and DON contamination and the percent presence of *F. poae* and NIV contamination were likewise statistically significant at values of *p* < 0.0001, *r* = 0.39 and *p* < 0.0001, *r* = 0.50, respectively. In addition, the correlation coefficient calculated as (total number of *F. graminearum* + *F. poae*/the presence of DON + NIV) was statistically significant (*p* < 0.0001, *r* = 0.40).

## 3. Discussion

The present work has provided novel data on the occurrence of *Fusarium* species and their toxins and has determined the two most frequently occurring species in barley, one of the most widely produced small-cereal grains in Argentina.

Although *F. graminearum* is currently considered the prevalent species in small-cereal grains, such as wheat and barley [16,33]; many studies have demonstrated a high frequency in the occurrence of other *Fusarium* species, such as *F. poae*, and have indicated that latter species as being significant and consequential in the ability to produce many mycotoxins whose deleterious effects, in general, have been well documented [34].

Two recent investigations analyzing the various FHB-producing species in malting barley in two different European regions, Italy and the UK, found that the occurrence of those species changed over the different years of the study [8,20]. In general, though, the main *Fusarium* species identified in barley grains were *F. avenaceum*, *F. graminearum*, *F. poae*, and *F. tricinctum* [8,20]. In the present work, we observed that barley grains harvested during 2012–2014 were contaminated mainly with *F. graminearum* and *F. poae*; and although the number of isolates of *F. graminearum* was higher than with *F. poae* (604 and 309, respectively), the percentage of samples was higher with *F. poae* than with *F. graminearum* (54.7% and 39.5%, respectively). That all barley genotypes were host to *F. graminearum* and/or *F. poae* is indeed notable. Although the *Fusarium*-species composition could vary from one year to the next, especially under different climatic conditions and between specific individual barley genotypes, as was subsequently verified by Beccari et al. [8]; the prevalent *Fusarium* species in the three years under the present study—it involving of the main regions of barley production in Argentina—were *F. graminearum* and *F. poae*. Nevertheless, since we did not have the same number of barley samples and/or localities per year, we were unable to validly compare separate years.

**Figure 4:**
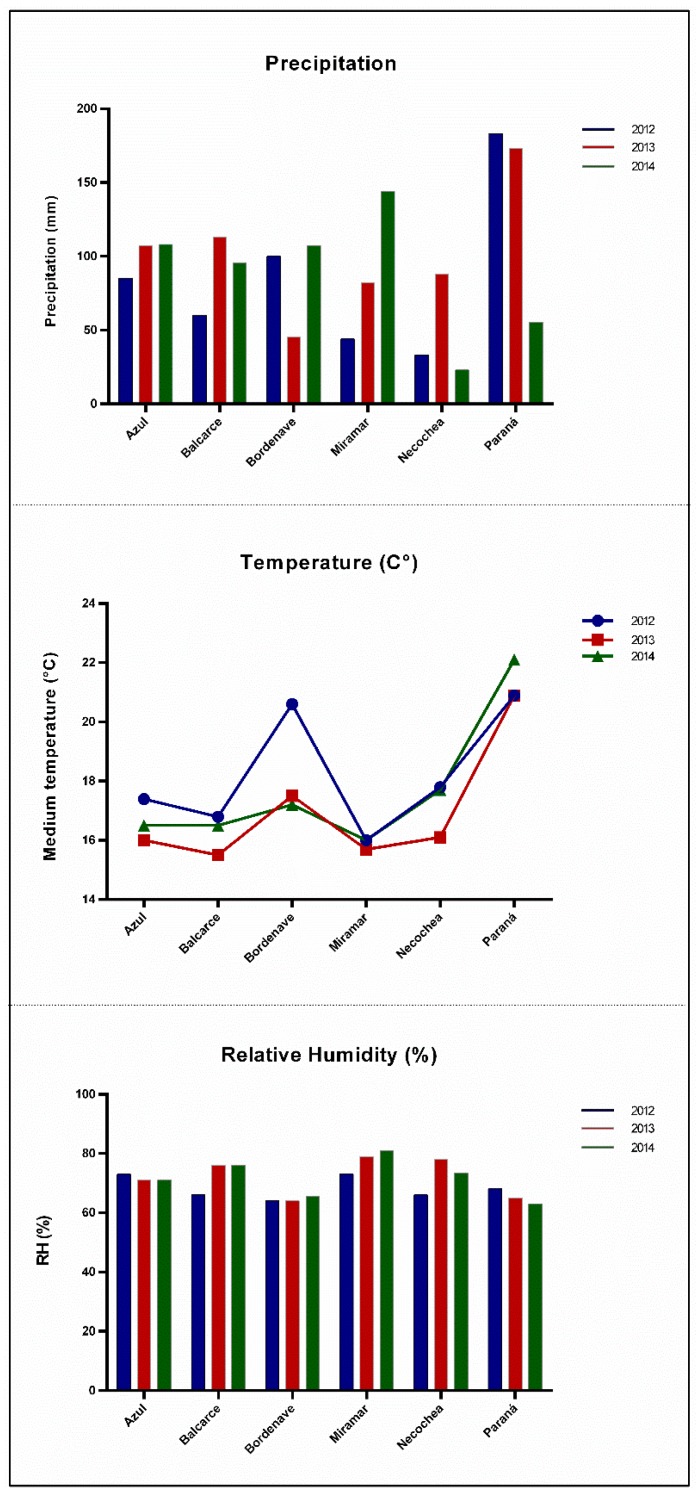
Accumulated precipitation, medium temperature and relative humidity (RH) at barley flowering period (15 October to 15 November) (*ordinate*). Localities were selected as representatives of geographical areas (*abscissa*). Color key indicate each sampled year: blue, 2012; red, 2013; and green, 2014.

In general, warm temperatures and wet conditions were found to favor *F. graminearum* infection, though temperatures around 25 °C and dryness did so for *F. poae* [35]. Table S1 and Figure 2 indicate that the areas sampled in certain instances were in close proximity but others were not. Moreover, the sowing dates had differed; and the general climatic conditions were variable (Figure 4), thus causing the average temperature and humidity during the barley’s flowering period to be different among separate localities. Apparently, these differing conditions within the sampled areas were insufficient to change the composition of the infective *Fusarium* species drastically, with *F. graminearum* and *F. poae* proving to be the best adapted species for colonization of the principal Argentine barley-growing areas. Nevertheless, a low number of other *Fusarium* species were found capable of producing trichothecenes, with the patchy occurrence of *F. pseudograminearum* and *F. cerealis* indicating the need for continuous monitoring.

The genomes of all the *F. graminearum* and *F. poae* screened for the potential production of DON and/or NIV became amplified positively for DON- and/or NIV-related sequences, as had been previously observed for Argentine isolates obtained from barley grains [29,30,34]. Therefore, since we isolated a high frequency of these *Fusarium* species from many samples, we could anticipate contaminations with these toxins as generated by those species. A correlation analyses accordingly confirmed those expectations. A comparable finding had been observed previously with respect to *F. graminearum* for DON and *F. poae* for NIV in wheat [18,22,36], barley [10,36], and oat [23]. Differences in the methodologies, however, can be cited that would be related to a different opportunity for species identification—i.e., DNA identification and/or quantification (e.g., qPCR) vs. conventional fungal isolation (where some species grow too slowly and are thus not recovered), and likewise with respect to the toxin detection and/or quantification methods.

Through the chemical analysis of the presence of DON and/or NIV, we documented that 45% of the samples contained mycotoxins, in varying concentrations and that NIV was the predominant one in those representative Argentine barley samples. By comparison, a study of Swiss barley revealed that the dominant species was *F. graminearum* and that DON was the most common mycotoxin, followed by NIV [10]. Nevertheless, although the levels of DNA were higher in *F. poae* than in *F. graminearum* in UK barley grains, the contamination with DON prevailed [20]. That European barley grains could be contaminated with another DON-producing species, but to our knowledge not yet isolated in Argentina—e.g., *F. culmorum*—is certainly relevant. In addition, different environmental conditions (such as climatic parameters) could possibly favor NIV over DON production in Argentine barley-growing areas.

As mentioned above, we were not able to survey the same number of samples per year and/or locality in the study, nevertheless the data in Figure 4 would indicate the influence of climatic conditions on the presence of *Fusarium* species and toxin contamination, consistent with the results from other studies [8,20]. For example, in Paraná (Entre Ríos province), where the agronomic practices were the same throughout the years of the study, the 2013 survey (Samples 65–70, Table S1) contained lower amounts of *Fusarium* and/or toxins than did the one in 2014 (Samples 116–119, Table S1). Moreover, the year 2014 in Paraná, occasioned higher amounts of NIV and *F. poae*, in concordance with dry conditions prevailing at the time (Figure 4). In contrast, certain samples presented both *F. graminearum* and *F. poae* and high amounts of DON and NIV (Samples 86 and 93, Table S1), thus enabling the possibility that general conditions for DON and/or NIV production were suitable and that different *Fusarium* species may be competing for the same specific niche so as to thus produce more toxins than if those species were in isolation. Furthermore, certain barley genotypes, but not all those analyzed, in Miramar (2013), contained different amounts of DON and *F. graminearum*, indicating—as was mentioned by Beccari et al. [8]—different responses between the barley genotype and the presence of *Fusarium* (Table S1).

In South America, and specifically Brazil and Uruguay, high amounts of DON were detected in malting-barley grains [26,27]. In our study, of the samples contaminated with DON, 12 (10%) contained levels higher than the European limits, and several also had high amounts of NIV. Argentina exports barley grains to different countries both with and without established trichothecene-toxin limits, while part of the production is used nationally in the beer industry, where maximum levels for DON and/or NIV have not yet been established.

The *Fusarium* biomass along with the *Fusarium* toxins, such as the trichothecenes DON and NIV, definitely alter the quality of malting [20,31,32], but the main concern regarding the occurrence of *Fusarium* species is that those same toxins are harmful to humans and animals and are furthermore widespread in all cereal-growing areas of the world. In the work reported here, we identified the main *Fusarium* species affecting natural malting-barley grains in Argentina and documented the presence of many samples with elevated concentrations of DON and NIV. Moreover, we detected samples containing both toxins. That limits on the concentration of both these toxins be established in barley grains in Argentina as soon as possible is therefore imperative.

Cereal-grain consumption is continuously increasing worldwide, which process will lead to an enhanced dependence on importation from developing countries. A real potential thus exists for both new and traditional exporters to remedy this imbalance, but the associated problems of food safety and environmental degradation must first be resolved [1]. With respect to food safety, a consideration of good agricultural practices, raw-material sanitation, and the conservation of natural resources—especially those renewable over those nonrenewable—is essential.

The information generated in the present study provides, for the first time, essential data on the *Fusarium* species and toxins associated with malting-barley grains in Argentina and thus contributes to the future development of sustainable-management strategies, such as plant resistance, sanitation, crop rotations, and the control of toxins in barley production.

## 4. Materials and Methods

### 4.1. Barley Samples and Climatic Information

A total of 119 grain barley samples were harvested and the grains supplied by producers from different areas where this crop had been sown during 2012 (22 samples), 2013 (61 samples), and 2014 (36 samples) (Table S1; Figure 2). The grain samples were reduced from 1 kg to 200 g with a grain divider and later reduced to 200 grains (10 g, approximately) for *Fusarium*-species isolation. Of the 200 g before reduction, 50 g were removed and maintained at 4 °C until analysis.

Climatic data (precipitation, temperature, and relative humidity) at the barley flowering period (15 October to 15 November) from representative localities (Azul, Balcarce, Bordenave, Miramar, Necochea and Paraná) were obtained from the National Meteorological Center (Figure 4). Except for 2014 in Bordenave, where precipitation of 182 mm was observed in a single day (26 October), in general, a normal regimen of precipitations was recorded for the different localities.

### 4.2. Fungal Isolation

The 200 seeds of each sample were surface-disinfected (70% *v*/*v* aqueous EtOH for 2 min; 5% (*w*/*v*) NaOCl for 2 min), rinsed twice in sterilized distilled water, and placed in Petri dishes (9 cm diam) with 2% (*w*/*v*) potato-dextrose agar (PDA) with chloramphenicol at 50 μg/mL and were incubated for approximately five days at 25 ± 2 °C in a 12 h light/dark photoperiod. Potential *Fusarium* isolates were transferred to 15-mL tubes with PDA and to Petri dishes with carnation-leaf-piece agar, for 7 days under the conditions described above. The isolates were morphologically identified by light microscopy according to the criteria of Leslie and Summerell [37] and were then stored at 4 °C on Spezieller–Nährstoffarmer agar.

### 4.3. Molecular Identification

Of the isolates identified as *F. graminearum* and *F. poae*, 10% were randomly selected (at least one isolate per sample) for DNA extraction by means of the cetyltrimethylammonium bromide method described by Stenglein and Balatti [38]. The quality of the DNA was verified by electrophoresis in 0.8% (*w*/*v*) agarose gels containing GelRed^TM^ (Biotium, Hayward, CA, USA) at 80 V in 1X Tris-borate-EDTA buffer for 1 h and visualized under ultraviolet light. The DNA concentration was estimated by fluorometery (Qubit^TM^ fluorometer, Invitrogen, Buenos Aires, Argentina).

The set of primers used for *F. graminearum* sensu stricto (later *F. graminearum*) identification were Fg16F: 5′-CTCCGGATATGTTGCGTCAA-3′ and Fg16R: 5′-GGTAGGTATCCGACATGGCAA-3′ [39], and for *F. poae* identification primers Fp82-F: 5′-CAAGCAAACAGGCTCTTCACC-3′ and Fp82-R: 5′-TGTTCCACCTCAGTGACAGGTT-3′ [40]. For the NIV and/or DON potential (genotypic characterization), the DNA of *F. graminearum* isolates was evaluated with primers based on the *Tri13* gene, Tri13NIVF: 5′-CCAAATCCGAAAACCGCAG-3′ and Tri13NIVR: 5′-TTGAAAGCTCCAATGTCGTG-3′; Tri13DONF: 5′-CATCATGAGACTTGTKCRAGTTTGGG-3′ and Tri13DONR: 5′-GCTAGATCGATTGTTGCATTGAG-3′ [41]. For the corresponding NIV potential, the DNA of *F. poae* isolates was evaluated with the primers designed from the *Tri7* gene, nivPf: 5′-TATCCTTGCATGGCAATGCC-3′ and nivPr: 5′-AAATGGCGATACGAGTATTGA-3′ [42].

The PCR reactions were performed after Parry and Nicholson [40], Nicholson et al. [39], Chandler et al. [41], and Dinolfo et al. [42]. Each PCR product was examined by gel electrophoresis along with positive and negative controls and a DNA-molecular-weight-standard ladder (100 bp), according to Castañares et al. [29]. DNA from *F. poae*, *F. graminearum* sensu stricto, *F. asiaticum*, *F. cortaderiae*, *F. meridionale*, and *F. austroamericanum* were used as controls for the different PCR reactions [29,42].

The results for a single isolate, selected at random, from each *Fusarium* species identified by morphological methods (*F. acuminatum*, *F. cerealis*, *F. chlamydosporum*, *F. equiseti-incarnatum*-species complex, *F. graminearum*, *F. oxysporum*, *F. poae*, *F. pseudograminearum*, *F. proliferatum* and *F. tricinctum*) were corroborated by sequencing the region of the elongation factor 1-α in comparison with the National Center for Biotechnology Information (NCBI) database web page [43].

### 4.4. Mycotoxin Analysis

Seeds (50 g) were finely ground in a blade homogenizer. An aliquot of 12.5 g of each ground sample was extracted with 50 mL of acetonitrile:water 86:16 (*v*/*v*) and shaken on an orbital shaker for 20 min. The supernatant was filtered through Nr. 101 fast-filter paper. For the clean-up, 8 mL were expressed through a Myco Sep Trich+^®^ column and 4 mL of the eluate evaporated to dryness under a flow of nitrogen at 60 °C on a Turbo Vap L work station (Caliper Life Sciences, Waltham, MA, USA). The resulting extracts were resuspended in 500 μL of the mobile phase and passed through a 0.22-μm microcellulose filter before injection into the HPLC-UV/Vis system.

The analysis was carried out with a Gilson HPLC-gradient-pump system equipped with a 151 UV/V is detector. The column was a 250 × 3-mm; 4-μm Hydro RP Phenomenex. The mobile phase was acetonitrile:water 20:80 running in the isocratic mode at 1 mL/min. The DON and NIV peaks were detected at 222 nm and the quantification was performed using external standards. The validation parameters as well as their acceptance criteria were in accordance with international guidelines [44,45]. The mycotoxins were quantified using an external standard. Linear range was between 0.156 and 2.5 µg/g, being 0.156 µg/g limit of quantification. Samples above linear range were diluted and re-analyzed to fit calibration curve values.

We used the criteria of the DON thresholds established by the European Commission for the evaluation of our results.

### 4.5. Statistical Analyses

The frequency of *Fusarium* species and toxin concentrations were analyzed by Pearson’s correlation coefficient with respect to the association between the toxin concentrations and the percentage of *F. graminearum* and/or *F. poae* identified in the barley samples. All the statistical analyses were performed with the InfoStat software (version 2016, InfoStat group UNC, Córdoba, Argentina, 2008) [46].

## Figures and Tables

**Table 1:** Percentages of samples with mycotoxins per year. Deoxynivalenol (DON); Nivalenol (NIV).

Year	2012	2013	2014
**% with DON**	9	13	47
**% with NIV**	27	18	47
**% without mycotoxins**	64	69	6

## Supplementary Materials

The following are available online at http://www.mdpi.com/2072-6651/10/2/78/s1. Table S1: Barley genotypes, localities, harvest years, mycotoxin concentration, and percentage of *F. graminearum* (Fg) and *F. poae* (Fp) isolates obtained per sample. Deoxynivalenol (DON); nivalenol (NIV).
