# Supplementary material for: Natural Contamination with Mycotoxins Produced by Fusarium graminearum and Fusarium poae in Malting Barley in Argentina
Source: Toxins (Basel). 2018 Feb 11;10(2):78. doi: 10.3390/toxins10020078 (PMC5848179; doi:10.3390/toxins10020078)
Supplement: Supplementary file 1 [file toxins-10-00078-s001.docx]

**Natural Contamination with Mycotoxins Produced by *Fusarium graminearum* and *Fusarium poae* in Malting Barley in Argentina**

María Soledad **Nogueira**, Julieta **Decundo,** Mauro **Martinez,** Susana Nelly **Dieguez,** Federico **Moreyra,** Maria Virginia **Moreno** and Sebastian Alberto **Stenglein**

**Table S1.** Barley genotypes, localities, harvest years, mycotoxin concentration, and percentage of *F. graminearum* (Fg) and *F. poae* (Fp) isolates obtained per sample. Deoxynivalenol (DON); nivalenol (NIV)

|  |  |  |  | **Trichothecene Concentration (μg/g)** | | **% *Fusarium*** | |
| --- | --- | --- | --- | --- | --- | --- | --- |
| **Sample** | **Barley Genotype** | **Locality** | **Province/Year** | **DON** | **NIV** | **Fg** | **Fp** |
| 1 | NN | T. Lauquen | Buenos Aires/2012 | n.d. | 0.31 | 2.00 | 3.00 |
| 2 | NN | Tres Arroyos | Buenos Aires/2012 | n.d. | 4.64 | n.d. | n.d. |
| 3 | Scarlett | Oliveros | Santa Fe/2012 | n.d. | n.d. | 1.00 | 1.50 |
| 4 | NN | Azul | Buenos Aires/2012 | n.d. | 0.54 | n.d. | n.d. |
| 5 | NN | Azul | Buenos Aires/2012 | 0.14 | n.d. | n.d. | 0.50 |
| 6 | Scarlett | T. Lauquen | Buenos Aires/2012 | n.d. | n.d. | 1.50 | 3.00 |
| 7 | NN | Azul | Buenos Aires/2012 | n.d. | n.d. | n.d. | n.d. |
| 8 | NN | Azul | Buenos Aires/2012 | n.d. | n.d. | n.d. | 0.50 |
| 9 | Scarlett | Azul | Buenos Aires/2012 | n.d. | n.d. | n.d. | 4.00 |
| 10 | Scarlett | T. Lauquen | Buenos Aires/2012 | 0.38 | 0.74 | 12.50 | 1.50 |
| 11 | Scarlett | Azul | Buenos Aires/2012 | n.d. | n.d. | n.d. | 0.50 |
| 12 | Scarlett | Azul | Buenos Aires/2012 | n.d. | 0.22 | n.d. | 1.50 |
| 13 | Scarlett | T. Lauquen | Buenos Aires/2012 | n.d. | n.d. | n.d. | n.d. |
| 14 | NN | Balcarce | Buenos Aires/2012 | n.d. | 0.33 | n.d. | 2.00 |
| 15 | NN | Balcarce | Buenos Aires/2012 | n.d. | n.d. | n.d. | 0.50 |
| 16 | NN | Loberia | Buenos Aires/2012 | n.d. | n.d. | 0.50 | 1.00 |
| 17 | NN | Loberia | Buenos Aires/2012 | n.d. | n.d. | n.d. | n.d. |
| 18 | NN | Quequen | Buenos Aires/2012 | n.d. | n.d. | n.d. | 1.00 |
| 19 | NN | Necochea | Buenos Aires/2012 | n.d. | n.d. | n.d. | n.d. |
| 20 | NN | San Cayetano | Buenos Aires/2012 | n.d. | n.d. | n.d. | n.d. |
| 21 | NN | Tres Arroyos | Buenos Aires/2012 | n.d. | n.d. | n.d. | 1.00 |
| 22 | NN | C. Pringles | Buenos Aires/2013 | n.d. | n.d. | n.d. | 0.50 |
| 23 | NN | C. Pringles | Buenos Aires/2013 | n.d. | n.d. | 0.50 | n.d. |
| 24 | NN | C. Suarez | Buenos Aires/2013 | n.d. | 0.70 | n.d. | 2.00 |
| 25 | NN | La Madrid | Buenos Aires/2013 | n.d. | 3.42 | n.d. | 23.00 |
| 26 | NN | Azul | Buenos Aires/2013 | n.d. | n.d. | n.d. | 0.50 |
| 27 | NN | Azul | Buenos Aires/2013 | n.d. | n.d. | 1.00 | n.d. |
| 28 | Andreia | Tandil | Buenos Aires/2013 | n.d. | n.d. | n.d. | n.d. |
| 29 | NN | Azul | Buenos Aires/2013 | n.d. | n.d. | n.d. | n.d. |
| 30 | Scarlett | Necochea | Buenos Aires/2013 | n.d. | 0.10 | n.d. | 0.50 |
| 31 | NN | Azul | Buenos Aires/2013 | n.d. | n.d. | 2.50 | 1.00 |
| 32 | NN | Tandil | Buenos Aires/2013 | n.d. | n.d. | 0.50 | 1.00 |
| 33 | NN | Balcarce | Buenos Aires/2013 | n.d. | n.d. | 1.50 | n.d. |
| 34 | NN | Balcarce | Buenos Aires/2013 | 0.89 | n.d. | 10.00 | n.d. |
| 35 | NN | Loberia | Buenos Aires/2013 | n.d. | n.d. | 3.00 | n.d. |
| 36 | NN | Loberia | Buenos Aires/2013 | n.d. | 0.94 | 2.00 | 0.50 |
| 37 | NN | Necochea | Buenos Aires/2013 | n.d. | n.d. | 2.00 | 0.50 |
| 38 | NN | Necochea | Buenos Aires/2013 | n.d. | 0.10 | 0.50 | n.d. |
| 39 | NN | San Cayetano | Buenos Aires/2013 | n.d. | n.d. | n.d. | 0.50 |
| 40 | NN | Tres Arroyos | Buenos Aires/2013 | n.d. | n.d. | n.d. | 11.50 |
| 41 | NN | C. Pringles | Buenos Aires/2013 | n.d. | n.d. | n.d. | n.d. |
| 42 | NN | C. Pringles | Buenos Aires/2013 | n.d. | n.d. | n.d. | n.d. |
| 43 | NN | C. Suarez | Buenos Aires/2013 | n.d. | n.d. | n.d. | n.d. |
| 44 | NN | Saavedra | Buenos Aires/2013 | n.d. | 5.78 | n.d. | n.d. |
| 45 | NN | Guamini | Buenos Aires/2013 | n.d. | n.d. | n.d. | 0.50 |
| 46 | NN | Daireaux | Buenos Aires/2013 | n.d. | n.d. | n.d. | 1.00 |
| 47 | NN | Olavarria | Buenos Aires/2013 | n.d. | 0.26 | 1.50 | 1.00 |
| 48 | Scarlett | T. Lauquen | Buenos Aires/2013 | n.d. | n.d. | 1.00 | n.d. |
| 49 | Andreia | NN | NN/2013 | n.d. | 0.49 | n.d. | n.d. |
| 50 | Andreia | NN | NN/2013 | n.d. | 0.62 | n.d. | n.d. |
| 51 | Scarlett | NN | NN/2013 | n.d. | n.d. | n.d. | 1.50 |
| 52 | Scarlett | NN | NN/2013 | n.d. | n.d. | n.d. | n.d. |
| 53 | Explorer | Bordenave | Buenos Aires/2013 | n.d. | n.d. | n.d. | n.d. |
| 54 | Scrabble | Bordenave | Buenos Aires/2013 | n.d. | n.d. | n.d. | n.d. |
| 55 | Shakira | Bordenave | Buenos Aires/2013 | n.d. | n.d. | n.d. | n.d. |
| 56 | Andreia | Bordenave | Buenos Aires/2013 | n.d. | n.d. | n.d. | n.d. |
| 57 | Carisma | Bordenave | Buenos Aires/2013 | n.d. | n.d. | n.d. | n.d. |
| 58 | Scarlett | Bordenave | Buenos Aires/2013 | 0.91 | n.d. | 0.50 | 1.50 |
| 59 | Explorer | Miramar | Buenos Aires/2013 | 0.27 | n.d. | 6.50 | n.d. |
| 60 | Scrabble | Miramar | Buenos Aires/2013 | 0.99 | n.d. | 1.50 | n.d. |
| 61 | Shakira | Miramar | Buenos Aires/2013 | n.d. | n.d. | n.d. | n.d. |
| 62 | Andreia | Miramar | Buenos Aires/2013 | 12.01 | n.d. | 3.50 | n.d. |
| 63 | Carisma | Miramar | Buenos Aires/2013 | 1.35 | n.d. | 6.50 | n.d. |
| 64 | Scarlett | Miramar | Buenos Aires/2013 | n.d. | n.d. | 2.00 | n.d. |
| 65 | Explorer | Paraná | Entre Rios/2013 | n.d. | n.d. | n.d. | n.d. |
| 66 | Scrabble | Paraná | Entre Rios/2013 | 0.16 | n.d. | n.d. | n.d. |
| 67 | Shakira | Paraná | Entre Rios/2013 | 0.20 | 0.85 | n.d. | n.d. |
| 68 | Andreia | Paraná | Entre Rios/2013 | n.d. | n.d. | n.d. | n.d. |
| 69 | Carisma | Paraná | Entre Rios/2013 | n.d. | n.d. | 0.50 | 0.50 |
| 70 | Scarlett | Paraná | Entre Rios/2013 | n.d. | n.d. | n.d. | n.d. |
| 71 | Explorer | Dorrego | Buenos Aires/2013 | n.d. | n.d. | n.d. | n.d. |
| 72 | Scrabble | Dorrego | Buenos Aires/2013 | n.d. | n.d. | 0.50 | 0.50 |
| 73 | Shakira | Dorrego | Buenos Aires/2013 | n.d. | n.d. | n.d. | 0.50 |
| 74 | Andreia | Dorrego | Buenos Aires/2013 | n.d. | n.d. | n.d. | 1.00 |
| 75 | Carisma | Dorrego | Buenos Aires/2013 | n.d. | 6.25 | n.d. | 1.00 |
| 76 | Scarlett | Dorrego | Buenos Aires/2013 | n.d. | n.d. | n.d. | 1.00 |
| 77 | Explorer | 9 de Julio | Buenos Aires/2013 | n.d. | n.d. | 1.00 | 0.50 |
| 78 | Scrabble | 9 de Julio | Buenos Aires/2013 | n.d. | n.d. | n.d. | n.d. |
| 79 | Shakira | 9 de Julio | Buenos Aires/2013 | n.d. | n.d. | n.d. | n.d. |
| 80 | Carisma | 9 de Julio | Buenos Aires/2013 | n.d. | n.d. | 1.00 | 0.50 |
| 81 | Scarlett | 9 de Julio | Buenos Aires/2013 | n.d. | n.d. | n.d. | n.d. |
| 82 | Scarlett | Azul | Buenos Aires/2013 | n.d. | n.d. | n.d. | n.d. |
| 83 | Andreia | Azul | Buenos Aires/2014 | n.d. | n.d. | 0.50 | n.d. |
| 84 | Scarlett | NN | NN/2014 | n.d. | n.d. | n.d. | 2.00 |
| 85 | NN | Tandil | Buenos Aires/2014 | n.d. | 5.13 | 10.00 | 7.00 |
| 86 | NN | Tandil | Buenos Aires/2014 | 1.34 | 4.10 | 28.50 | 2.50 |
| 87 | NN | Ayacucho | Buenos Aires/2014 | 2.20 | n.d. | 19.00 | 3.00 |
| 88 | NN | Ayacucho | Buenos Aires/2014 | 1.26 | n.d. | 6.50 | 4.50 |
| 89 | NN | Ayacucho | Buenos Aires/2014 | 1.22 | n.d. | 31.00 | 0.50 |
| 90 | NN | Ayacucho | Buenos Aires/2014 | 3.74 | 0.36 | 17.50 | 0.50 |
| 91 | NN | Ayacucho | Buenos Aires/2014 | 5.04 | n.d. | 29.50 | 0.50 |
| 92 | NN | Balcarce | Buenos Aires/2014 | n.d. | n.d. | 8.00 | 0.50 |
| 93 | NN | Balcarce | Buenos Aires/2014 | 5.32 | 4.00 | 23.00 | 0.50 |
| 94 | NN | Loberia | Buenos Aires/2014 | 7.48 | 1.30 | 22.00 | n.d. |
| 95 | NN | Loberia | Buenos Aires/2014 | 0.24 | 0.20 | 9.50 | n.d. |
| 96 | NN | Loberia | Buenos Aires/2014 | 0.40 | n.d. | 9.00 | 1.00 |
| 97 | NN | Quequen | Buenos Aires/2014 | 0.24 | n.d. | 6.00 | 0.50 |
| 98 | NN | Loberia | Buenos Aires/2014 | 0.74 | n.d. | 7.50 | n.d. |
| 99 | NN | Necochea | Buenos Aires/2014 | 0.51 | 0.75 | 3.50 | 2.00 |
| 100 | NN | San Cayetano | Buenos Aires/2014 | n.d. | 4.58 | 1.50 | 6.00 |
| 101 | NN | Tres Arroyos | Buenos Aires/2014 | n.d. | n.d. | 0.50 | 1.50 |
| 102 | NN | C. Pringles | Buenos Aires/2014 | n.d. | 0.42 | n.d. | n.d. |
| 103 | NN | C. Suarez | Buenos Aires/2014 | n.d. | n.d. | n.d. | 1.00 |
| 104 | NN | C. Suarez | Buenos Aires/2014 | n.d. | n.d. | n.d. | 1.00 |
| 105 | NN | Guamini | Buenos Aires/2014 | n.d. | 4.61 | n.d. | 3.00 |
| 106 | NN | C. Suarez | Buenos Aires/2014 | n.d. | n.d. | 0.50 | 3.50 |
| 107 | NN | Guamini | Buenos Aires/2014 | n.d. | n.d. | 1.00 | 1.00 |
| 108 | NN | Bolivar | Buenos Aires/2014 | n.d. | 4.64 | n.d. | 3.50 |
| 109 | NN | Bolivar | Buenos Aires/2014 | 9.53 | n.d. | n.d. | 2.50 |
| 110 | Scarlett | NN | Buenos Aires/2014 | n.d. | 2.17 | n.d. | n.d. |
| 111 | Andreia | NN | Buenos Aires/2014 | 7.35 | n.d. | n.d. | n.d. |
| 112 | Andreia | NN | NN/2014 | 0.71 | n.d. | n.d. | n.d. |
| 113 | Explorer | Bordenave | Buenos Aires/2014 | 6.29 | n.d. | n.d. | n.d. |
| 114 | Scarlett | Bordenave | Buenos Aires/2014 | n.d. | n.d. | n.d. | n.d. |
| 115 | Scarlett | Miramar | Buenos Aires/2014 | n.d. | 2.63 | n.d. | 0.50 |
| 116 | Andreia | Paraná | Entre Rios/2014 | n.d. | 4.80 | n.d. | 11.50 |
| 117 | Explorer | Paraná | Entre Rios/2014 | n.d. | 7.71 | n.d. | 10.00 |
| 118 | Scarlett | Paraná | Entre Rios/2014 | n.d. | 0.59 | n.d. | 2.00 |
| 119 | Scrabble | Paraná | Entre Rios/2014 | n.d. | 5.81 | n.d. | 8.50 |

NN = data not available; n.d. = not detecte d.
